# Induction of apoptosis in human cancer cell lines by the novel anthracenyl-amino acid topoisomerase I inhibitor NU/ICRF 505.

**DOI:** 10.1038/bjc.1996.368

**Published:** 1996-08

**Authors:** I. Meikle, J. Cummings, J. S. Macpherson, J. F. Smyth

**Affiliations:** Imperial Cancer Research Fund Medical Oncology Unit, Western General Hospital, Edinburgh, UK.

## Abstract

**Images:**


					
Bridsh Journal of Cancer (1996) 74, 374-379
? 1996 Stockton Press All rights reserved 0007-0920/96 $12.00

Induction of apoptosis in human cancer cell lines by the novel anthracenyl-
amino acid topoisomerase I inhibitor NU/ICRF 505

I Meikle, J Cummings, JS Macpherson and JF Smyth

Imperial Cancer Research Fund Medical Oncology Unit, Western General Hospital, Edinburgh EH4 2XU, UK.

Summary Anthracenyl-amino acid conjugates represent a novel chemical class of topoisomerase (topo)
inhibitor. NU/ICRF 505 is a lead compound that stabilises topo I cleavable complexes and is actively cytotoxic
at low MM concentrations. In this study, endonucleolytic DNA cleavage was used as a marker of apoptosis to
investigate mechanisms of cell death produced by this compound. NU/ICRF 505 (5 yM) induced a substantial
increase in the level of DNA fragmentation in HL60 cells (up to 30% of total extracted DNA) but only after a
48 and 72 h drug exposure (compared with 6 h after treatment with camptothecin), as determined qualitatively
by conventional gel electrophoresis and quantitatively by spectrofluorimetry. This effect was substantially
reversed by co-treatment with zinc (1 mM). Subsequent studies with the human lung (NX002), ovarian (A2780)
and colon (HT29) cancer cell lines yielded evidence of formation of higher molecular weight DNA fragments in
NX002 and A2780 cells in response to NU/ICRF 505 (5 uM). Co-treatment with zinc (1 mM) caused a small
decrease in DNA fragmentation. These data suggest that the induction of apoptosis may play an important
role in the mechanism of cytotoxicity of NU/ICRF 505 in HL60 cells and that other pathways of cell death
may also be operative in NX002 and A2780 in conjunction with apoptosis.

Keywords: anthracenyl-amino acid; NU/ICRF 505; topoisomerase inhibitor; apoptosis; gel electrophoresis;
human cell line

DNA topoisomerases I and II (topo I and II) are nuclear
enzymes that play critical roles in DNA metabolism (Wang,
1985) and appear to be primary targets for a number of
clinically active anti-cancer drugs (Schneider et al., 1990;
Corbett and Osheroff, 1993; Chen and Liu, 1994). Drugs
inhibit these enzymes by stabilising a reaction intermediate,
referred to as the 'cleavable complex', where the enzyme
remains covalently bound to DNA after strand cleavage
(Nelson et al., 1984; Hsiang et al., 1985; Cummings and
Smyth, 1993), and there has been much debate as to how
such complexes induce cytotoxicity (Zwelling, 1989; Gewirtz
et al., 1991; Bertrand et al., 1991). Studies are beginning to
focus on the link between topo inhibition and the induction
of programmed cell death (or 'apoptosis') (Walker et al.,
1991; Bertrand et al., 1991; Hickman, 1992; Onishi et al.,
1993), a process which is usually recognised morphologically
by a marked condensation of chromatin and shrinkage of the
nucleus (Wyllie et al., 1980, 1984; Earnshaw, 1995). However,
apoptosis is often characterised by endonucleolytic cleavage
of DNA, which can be viewed by electrophoresis as a series
of bands, or 'DNA ladder' (Wyllie, 1980; Arends et al.,
1990). This particular approach has been employed with a
number of topo inhibitors to determine the presence of
apoptosis (Kaufmann, 1989; Walker et al., 1991; Roy et al.,
1992; Gulliya et al., 1994; Huschtscha et al., 1995). DNA
laddering, which can be reversed by zinc (Cohen and Duke,
1984), may not be an ideal marker for apoptosis as there
have been a number of reports describing the morphological
characteristics of apoptotic cells without internucleosomal
cleavage (Cohen et al., 1992; Oberhammer et al., 1993a; Sun
et al., 1994). However, it has been reported recently that
during early apoptosis DNA is cleaved into larger fragments
which may serve as precursors to the smaller oligonucleoso-
mal fragments (Bicknell et al., 1994).

Many recently described topo I and II inhibitors have
been demonstrated not to stabilise the cleavable complex but
to act on the catalytic activity of the enzyme (Cummings and

Smyth, 1993; Li et al., 1993; Permana et al., 1994). These
catalytic inhibitors can also display substantial levels of in
vitro cytotoxicity (Ishida et al., 1991). Although a number of
unique mechanisms of action have been proposed to explain
catalytic inhibition of topo I and II, there have been reports
that these agents can also induce endonucleolytic DNA
cleavage (Onishi et al., 1993; Markovits et al., 1994; Onishi et
al., 1994).

Anthracenyl-amino acid conjugates (AACs) represent a
novel chemical class of topo inhibitor that can act either
through formation of the cleavable complex or by catalytic
inhibition (Meikle et al., 1995a) and are actively cytotoxic
against Chinese hamster ovary (CHO) and human cancer cell
lines at low gM concentrations (Cummings et al., 1994,
1995a; Meikle et al., 1995b). Recent studies using a
cytogenetic approach indicated that the topo I cleavage
inducer, NU/ICRF 505, and the topo II catalytic inhibitor,
NU/ICRF 500, also act as enzyme inhibitors in intact CHO
cells (Cummings et al., 1995b). As part of continuing
investigations into the mechanisms(s) of cell death induced
by these compounds, endonucleolytic DNA cleavage was
measured in HL60 human leukaemia cells exposed to NU/
ICRF 505 (see Figure 1 for structural details). In subsequent
experiments DNA fragmentation was assessed in a number of
human cancer cell lines: the ovarian cancer line, A2780; the

O     Amino acid

O     OH

Codename Amino acid C-terminus

NU/ICRF 505 Tyrosine

COOC2H5

Figure 1 Structure of the anthracenyl - amino acid conjugate
NU/ICRF 505. [2H,3H]-9, 10-dihydroxyanthracene-1, 4-dione
was reacted with a-tyrosine ethyl ester to produce the compound.

Correspondence: J Cummings

Received 13 November 1995; revised 26 February 1996; accepted 12
March 1996

Induction of apoptosis by NU/ICRF 505

I Meikle et al                                                        $

375

non-small-cell lung cancer line, NX002; and the colon cancer
line, HT29. By simultaneously determining cell number and
investigating the effects of zinc on levels of DNA
fragmentation induced by AACs, the findings in this study
are related to the possible induction of apoptosis.

Materials and methods
Materials

NU/ICRF 505 was synthesised and chemically characterised
as described in full in Cummings and Mincher, UK patent
Application Number GB 9205859.3; International Patent
Application Number PCT/GB93/00546. Camptothecin was
purchased from Sigma Chemical Co. (Poole, UK). Stock
solutions were made up fresh for each experiment in dimethyl
sulphoxide (DMSO) (spectroscopic grade) and the final
concentration of DMSO did not exceed 0.5% in cell culture
medium. Zinc, which was added as zinc sulphate, was
dissolved in sterile distilled water before addition to the
culture medium. The fluorescent stain, 4,6-diamidino-2-
phenylindole (DAPI), was purchased from Sigma, and the
123 bp ladder was purchased from Life Technologies, Paisley,
UK.

Cell culture studies

All cell lines were grown in RPMI-1640 medium
supplemented with 5% heat-inactivated fetal calf serum
containing a 1% antibiotic mixture under standard
conditions, and were maintained at 37?C in a humidified
atmosphere of 5% carbon dioxide in air. Cells were plated
in 175 cm2 flasks so as to achieve a final density of 1-
2 x 107 per flask (or 1 x 105 in some growth inhibition
studies) at time zero. At this time, medium was replaced
with either drug-containing medium, or medium containing
0.5% DMSO (control). At each time point, A2780, HT29
and NX002 cells were trypsinised before counting whereas
HL60 cells were harvested by centrifugation immediately.
Following two washes in phosphate-buffered saline (PBS),
200 p1 of each cell suspension was taken for the
determination of total cell number using a Coulter Counter
ZM (Coulter Electronics, Luton, UK). For later time points
in experiments involving the A2780, NX002 and HT29 cell
lines, the culture medium was also subjected to centrifuga-
tion to pellet detached cells, and these pellets were treated
as separate samples.

Results

Induction of DNA fragmentation in HL60 cells

Initial experiments investigated whether the topo I inhibitor
NU/ICRF 505 (see Figure 1 for structure) could induce
endonucleolytic DNA cleavage over a 72 h period in HL60
cells. Simultaneous determination of cell number during this
time period showed that NU/ICRF 505 at 5 gM caused a
marked reduction to a level similar to 0.1 gM camptothecin
(Figure 2). Using a quantitative fluorescence assay, over the
72 h study period camptothecin was demonstrated to induce
50% fragmentation of total extracted DNA compared with
30% for NU/ICRF 505 (Figure 3).

The degree of DNA fragmentation shown in Figure 3 was
confirmed by agarose gel electrophoresis of DNA extracts
(Figure 4). Camptothecin clearly produced characteristic
DNA laddering after only 4 h, which became progressively
more intense at 8 and 24 h (Figure 4, lanes 3-6). Similarly,

Co

0

x

a)

.0

E

C
C-

I

Time (h)

Figure 2 Total number of HL60 cells following exposure to
camptothecin or NU/ICRF 505. HL60 cells were exposed to
either 0.1 pM camptothecin or 5 pM NU/ICRF 505 for 2, 4, 8,
24, 48 and 72 h, and the total number of cells remaining at each
time point determined by Coulter Counting. Control cells were
incubated with fresh medium containing 0.5% DMSO. Results
represent a typical graph taken from experiments that were
repeated on four separate occasions. -Ol-, control; -U-, NU/
ICRF 505; -A-, camptothecin.

DNA extraction/gel electrophoresis

DNA was extracted from cell pellets using the nucleon II
DNA extraction kit manufactured by Scotlab Ltd (Coat-
bridge, UK). The final precipitated DNA fraction was left
to rehydrate in sterile distilled water at 4?C overnight, after
which time the concentration of DNA was determined
spectrophotometrically using a UV2 spectrometer manufac-
tured by ATI-Unicam (Cambridge, UK). DNA samples
were analysed by loading 4 pg of each sample onto a 1.8%
agarose gel in 40 mM Tris-acetate buffer (pH 8.0) containing
1 mM EDTA. Each gel also contained 1 pg ml-' ethidium
bromide to enable visualisation of the DNA bands on a
UV.-light box.

Quantitation of DNA fragmentation

Cell pellets, harvested as described above, were lysed in 0.5%
Triton X-100 containing 5 mM Tris-HCl (pH 7.4) and 1 mM
EDTA, for 20 min on ice. The percentage of DNA in the
supernatant following centrifugation of the lysate was
determined using the spectrofluorimetric method described
in detail by Onishi et al. (1993) in which a Baird Nova
spectrofluorimeter (Baird-Atomic Ltd, Braintree, UK) was
used to detect the fluorescence of each fraction following the
addition of DAPI.

C

0

C.

C

4)

E

0)

E
0

0

Time (h)

Figure 3 Quantitation of DNA fragmentation levels following
exposure of HL60 cells to camptothecin and NU/ICRF 505. As
for Figure 2, except at each time point the per cent DNA
fragmentation induced by each drug treatment was determined
spectrofluometrically as described in Materials and methods. -Cl-,
control; -o-, NU/ICRF 505; -A-, camptothecin.

Induction of apoptosis by NUAICRF 505

I Meikle et a!

NU/1CRF 505 produced a significant level of DNA laddering
but only after 48 h (Figure 4, lane 13) which increased at
72 h (Figure 4, lane 15).

Induction of DNA fragmentation in A2780 and NX0O2 cells

Following on from initial studies with HL60 cells, NU/1CRF
505 was used in further studies with three different human
cancer cell lines. A2780 and NX002 were both chemosensitive
to NU/1CRF 505 with 5 gum drug producing a similar effect
on cell counts to 0.1 gIm camptothecin (illustrated only for
A2780 in Figure 5). However, these effects were possibly not

Figure 4 DNA fragmentation in HL60 cells exposed to
camptothecin and NU/ICRF 505. As for Figure 2 except that
the cell pellets obtained at each time point were used to extract
total cell DNA. DNA extracts (4 Mig each) were subjected to gel
electrophoresis on 1.8% agarose gels as described in Materials
and methods with the following gel loadings: lane 1, 123 bp DNA
ladder; lane 2, 0 h control; lanes 3 -6, 2, 4, 8 and 24 h
camptothecin exposure respectively; lane 7, 24 h control; lanes
8 -11, 2, 4, 8 and 24 h NU/ICRF 505 exposure respectively; lane
12, 48 h control; lane 13, 48 h NU/ICRF 505; lane 14, 72 h
control; lane 15, 72 h NU/ICRF 505. Results represent a typical
example for n = 3 replicates.

x
0)

-0) '

0  1

as dramatic as in the HL60 cell line (compare with Figure 2).
Experiments with the human non-small-cell lung cancer cell
line, NX002, revealed evidence of high molecular weight
DNA fragmentation in those cells detached from culture
flasks at 24, 48 and 72 h (Figure 6a, lanes 6, 8 and 10
respectively). Those cells remaining attached to culture flasks
did not show any evidence of fragmentation (Figure 6a, lanes
3 -5, 7 and 9). The medium from 72 h control cells did not
contain a sufficiently high number of detached cells to enable
extraction of DNA for electrophoresis, although cells
remaining attached to the flask at this time did not show
noticeable DNA fragmentation (Figure 6a and b, lane 1 1). A

a

1    2   3    4   5    6    7   3    9   1 0  1 1

b

1   2  3   4   5  6   7      9   1 0 1 1

I)          20          401

Time (h)

60U8

Figure 5 Total number of A2780 cells following exposure to
camptothecin and NU/ICRF 505. Cells were exposed to either
0.1 pM camptothecin or 5 MM NU/ICRF 505 for 6, 24, 48 and
72 h, and the total number of cells remaining at each time point
determined by Coulter Counting. Control cells were incubated
with fresh medium containing 0.5% DMSO. Results represent a
typical graph taken from experiments that were repeated on four
separate occasions. -El-, control; -E-, NU/ICRF 505; -A-,
camptothecin.

Figure 6 DNA fragmentation in human non-small-cell lung and
ovarian cancer cells exposed to NU/ICRF 505. DNA was
extracted from cells following various exposure times to NU/
ICRF 505 and analysed by gel electrophoresis as described in
Materials and methods. Upper gel (a), NX002 lung cancer cells;
lower gel (b), A2780 ovarian cancer cells. Gel loadings as follows
(4 Mig per sample): lane 1, 123 bp ladder; lane 2, 0 h control; lanes
3, 4, 5, 7 and 9, DNA from attached cells following 4, 8, 24, 48
and 72 h exposure to NU/ICRF 505; lanes 6, 8 and 10, DNA
from detached cells following 24, 48 and 72 h exposure to NU/
ICRF 505; lane 11, 72 h control. Results represent a typical
example for n =3 replicates.

9

376

&A -

t
E

1

n

U,N.-       ..      - -        . ..      ..........    .......                    . ..  .. ...   ...

.... .........

.. ..... .... ..... .
t         -tF --E

I M.Off                                                                                               zz,

........         .................

........        ...................

Induction of apoptosis by NU/ICRF 505
I Meikle et al

very similar pattern was obtained with the human ovarian
cancer cell line, A2780. Cell pellets obtained from centrifuga-
tion of the culture medium at 24, 48 and 72 h yielded
evidence of high molecular weight DNA fragmentation
(Figure 6b, lanes 6, 8 and 10 respectively).

The HT29 colon cancer cell line was chemoresistant to
NU/ICRF 505 at 5 ,M and, correspondingly, no DNA
fragmentation was apparent at any of the time points studied
(results not shown).

Possibly owing to the high molecular weight nature of the
DNA fragmentation observed, the spectrofluorimetric meth-
od used to quantitate levels of DNA fragmentation in HL60
cells was not sufficiently sensitive to give reliable values with
the NX002 and A2780 cell lines. This method was
consequently not used in studies with these cell lines.

Effect of zinc on DNA fragmentation and cell number in HL60,
A2780 and NX002 cells

The values for cell counts at 48 h showed that the addition of
zinc substantially enhanced the survival of HL60 cells
exposed to both 1 pM camptothecin (23.7% vs 1.6%) and
20 gIM NU/ICRF 505 (17.7% vs 3.3%). This was confirmed
in Figure 7 (lanes 11 and 12) in which the level of DNA
fragmentation was clearly reduced in the zinc-treated cells
(camptothecin-treated samples are not shown in this figure as
there was an insufficient number of cells present at this time
for DNA extraction). The effect of zinc addition on cell
numbers in A2780 and NX002 was less marked, where both
camptothecin and NU/ICRF 505 showed similar small
increases in survival (3.2-4.9% increase). Analysis of DNA
from zinc-treated NX002 cells revealed a reduction in the
level of DNA fragmentation in camptothecin-treated cells
(Figure 7, lanes 7 and 8), which was also apparent in NU/
ICRF 505-treated cells (Figure 7, lanes 9 and 10). These
effdcts were less obvious in A2780 cells (Figure 7).

Discussion

The aim of the present study has been to determine whether
or not anthracenyl-amino acid conjugates, a new chemical
class of topo inhibitor, can induce apoptosis through
endonucleolytic cleavage of DNA. One of the most
promising candidates from this class was investigated: NU/
ICRF 505, which stabilises topo I cleavable complexes
(Meikle et al., 1995a). Initial experiments were carried out
in HL60 human promyelocytic leukaemia cells as previous
reports have demonstrated that this line exhibits particularly
high levels of DNA fragmentation in response to known topo
inhibitors (Kaufmann, 1989; Del Bino et al., 1991; Solary et
al., 1993) without the relatively high levels of spontaneous
DNA fragmentation observed in untreated thymocytes
(Wyllie, 1980; Onishi et al., 1993).

Using camptothecin as a positive control, characteristic
DNA laddering was detected following only a 4 h drug
exposure and this is consistent with previous reports
(Kaufmann, 1989). Characteristic DNA laddering was also
detected with NU/ICRF 505 in HL60 cells - suggesting that
this compound can also induce apoptosis - but only after a
48 h drug exposure. Such a long delay and the reduced level
of DNA fragmentation may be explained by the fact that
NU/ICRF 505 is less effective at stabilising topo I cleavable
complexes compared with camptothecin (Meikle et al.,
1995a).

Treatment of HL60 cells with zinc (which is known to
antagonise endonuclease activation; Cohen and Duke, 1984)

appeared to markedly enhance cell survival to both
camptothecin and NU/ICRF 505. In addition, this was
associated with a clear reduction in the level of DNA
fragmentation providing further evidence that NU/ICRF 505
induces programmed cell death in HL60 cells.

Following on from the findings with HL60 cells, a number
of other human cancer cell lines were treated with NU/ICRF

1   2     3   4    5   6

7   8

9   10    11  12

Figure 7 DNA fragmentation in human cancer cells co-treated
with zinc and camptothecin or NU/ICRF 505. A2780, NX002
and HL60 cells were either exposed to 20 ,uM NU/ICRF 505 or
1 iM camptothecin alone or in the presence of 1 mM zinc
sulphate. Following 48 h drug exposure, total cell DNA was
extracted and analysed by agarose gel electrophoresis as described
in Materials and methods. Sample identification as follows: lane
1, 123 bp ladder; lane 2, 48 h control; lanes 3 and 5, A2780 cells
exposed to camptothecin and NU/ICRF 505 respectively; lanes 4
and 6, as for 3 and 5 except in the presence of zinc; lanes 7 and 9,
NX002 cells exposed to camptothecin and NU/ICRF 505
respectively; lanes 8 and 10, as for 7 and 9 except in the
presence of zinc; lane 11, HL60 cells exposed to NU/ICRF 505;
lane 12, as for lane 11 except in the presence of zinc.

505 in order to determine levels of DNA fragmentation. Both
the non-small-cell lung cancer cell line, NX002, and the
ovarian cancer cell line, A2780, responded with a marked
reduction in cell number after 72 h. Although there was an
absence of distinct low molecular weight nucleosomal size
DNA fragments, there was evidence on gels of higher
molecular weight DNA fragmentation. Importantly, this
effect was observed only in those cells which had detached
from the culture flasks. There have now been several recent
reports recognising that the formation of high molecular
weight DNA can arise as a consequence of treatment of cells
with topo I and II inhibitors (Fillipski et al., 1990; Bicknell et
al., 1994; Sun et al., 1994; Cohen et al., 1994). The formation
of high molecular weight DNA is of particular significance as
this can occur in cases of apoptosis in which low molecular
weight internucleosomal DNA cleavage is absent (Oberham-
mer, 1993b). However, during apoptosis large fragments are
thought to be formed initially, and it is from these that the
characteristic DNA ladders are derived (Cohen et al., 1994).
The apparent appearance of only higher molecular weight
fragments following prolonged periods of drug exposure
(after 24 and 48 h for example) in the A2780 and NX002 cells
reported here, however, suggests that this transition did not
appear to occur in these two lines, although both do show a
noticeable increase in smaller DNA fragments following the
72 h drug exposure. The technique of pulsed-field gel
electrophoresis has been applied in the analysis of high
molecular weight DNA fragments formed during apoptosis
(Walker et al., 1991; Roy et al., 1992) and this would be of
value in the present study to determine if these fragments can
be resolved into the distinct species characteristic of apoptosis
(Cohen et al., 1994).

High molecular weight DNA smearing has also been
ascribed to cells undergoing necrosis (Huschtscha et al.,
1995), and experiments were performed in which A2780 and
NX002 cells were co-treated with NU/ICRF 505 and zinc in
an initial attempt to distinguish between the two different
mechanisms (Cohen and Duke, 1984). The small increase in
cell number in the presence of zinc for both camptothecin
and NU/ICRF 505 corresponded well with a small, though
noticeable, reduction in levels of DNA fragmentation in both

2                   s

-f

I

s:t

::: : : M :?
: : : : :44 : : M :?

:: ... : :

A:

Induction of apoptosis by NU/ICRF 505

I Meikle et al

07Q

cell lines. Consequently, unlike the equivalent study with
HL60 cells described above, the results of zinc co-treatment
of A2780 and NX002 cells are much less obvious.
Endonuclease activation is possibly not the sole cause of
DNA fragmentation in these cell lines and other mechanisms
of cytotoxicity are now also under consideration.

In summary, we have employed endonucleolytic DNA
fragmentation as a marker to provide evidence that the AAC
topo I cleavage inducer, NU/ICRF 505, induces apoptosis in

References

ARENDS MJ, MORRIS RG AND WYLLIE AH. (1990). Apoptosis. The

role of the endonuclease. Am. J. Pathol., 136, 593 -608.

BERTRAND R, KERRIGAN D, SARANG M AND POMMIER Y. (1991).

Cell death induced by topoisomerase inhibitors. Role of calcium
in mammalian cells. Biochem. Pharmacol., 42, 77 - 85.

BICKNELL GR, SNOWDEN RT AND COHEN GM. (1994). Formation

of high molecular mass DNA fragments is a marker of apoptosis
in the human leukaemic cell line, U937. J. Cell Sci., 107, 2483-
2489.

CHEN AY AND LIU LF. (1994). DNA topoisomerase: essential

enzymes and lethal targets. Annu. Rev. Pharmacol. Toxicol., 34,
191 -218.

COHEN GM, SUN XM, SNOWDEN RT, DINSDALE D AND

SKILLETER DN. (1992). Key morphological features of apoptosis
may occur in the absence of internucleosomal DNA fragmenta-
tion. Biochem. J., 286, 331-334.

COHEN GM, SUN XM, FEARNHEAD H, MACFARLANE M, BROWN

DG, SNOWDEN RT AND DINSDALE D. (1994). Formation of large
molecular weight fragments of DNA is a key committed step of
apoptosis in thymocytes. J. Immunol., 153, 507-516.

COHEN JJ AND DUKE RC. (1984). Glucocorticoid activation of a

calcium-dependent endonuclease in thymocyte nuclei leads to cell
death. J. Immunol., 132, 38-42.

CORBETT AH AND OSHEROFF N. (1993). When good enzymes go

bad: conversion of topoisomerase II to a cellular toxin by
antineoplastic drugs. Chem. Res. Toxicol., 6, 585- 597.

CUMMINGS J AND SMYTH JF. (1993). DNA topoisomerase I and II

as targets for rational design of new anticancer drugs. Ann.
Oncol., 4, 533-543.

CUMMINGS J, MACPHERSON JS, HICKSON ID, DAVIES SL,

MINCHER DJ AND SMYTH JF. (1994). Drug development of
anthracenyl -amino acid/peptide topoisomerase I and II inhibi-
tors which circumvent drug resistance. Ann. Oncol., 5, (suppl. 5),
186.

CUMMINGS J, MACPHERSON JS, MEIKLE I AND SMYTH JF. (1995a).

Development of anthracenyl- amino acid/peptide topoisomerase
I and II inhibitors which circumvent drug resistance. Biochem.
Pharmacol., (in press).

CUMMINGS J, SUMMER AT, SLAVOTINEK A, MEIKLE I, MAC-

PHERSON JS AND SMYTH JF. (1995b). Cytogenetic evaluation of
the mechanism of cell death induced by the novel anthracenyl-
amino acid topoisomerase II catalytic inhibitor NU/ICRF 500.
Mutat. Res., 344, 55-62.

DEL BINO G AND DARZYNKIEWICZ Z. (1991). Camptothecin,

teniposide, or 4'-(9-acridinylamino)-3-methanesulfon-m-anisi-
dide, but not mitoxantrone or doxorubicin, induces degradation
of nuclear DNA in the S phase of HL60 cells. Cancer Res., 51,
1165-1169.

EARNSHAW WC. (1995). Nuclear changes in apoptosis. Curr. Opin.

Cell Biol., 7, 337-343.

FILLIPSKI J, LEBLANC J, YOUDALE T, SIKORSKA M AND WALKER

PR. (1990). Periodicity of DNA folding in higher order chromatin
structures. EMBO J., 9, 1319- 1327.

GULLIYA KS, FRANCK B, SCHNEIDER U, SHARMA R, ARNOLD L

AND MATTHEWS JL. (1994). Topoisomerase II-dependent novel
antitumour compounds merocil and merodantoin induce apop-
tosis in Daudi cells. Anticancer Drugs, 5, 557- 566.

GEWIRTZ DA. (1991). Does bulk damage to DNA explain the

cytotoxic effects of topoisomerase inhibitors? Biochem. Pharma-
cot., 42, 2253-2258.

HICKMAN IA. ( 1992). Apoptosis induced by anticancer drugs.

Cancer Metastasis Rev., 11, 121 -139.

HSIANG Y.-H, HERTZBERG R, HECHT S AND LIU LF. ( 1985).

Camptothecin induces protein-linked DNA breaks via mamma-
lian DNA topoisomerase I. J. Biol. Chem., 260, 14873- 14878.

HL60 cells. Similar investigations in human lung and ovarian
cancer cells suggest apoptosis may participate in the
induction of cytotoxicity in these lines, however, additional
work is required to clarify its precise role. These results
provide important information on mechanisms of cell death
induced by this class of compound and will be of benefit in
the further development of AACs as potential anti-cancer
drugs exhibiting novel properties.

HUSCHTSCHA LI, BARTIER WA, MALMSTROM A AND TATTER-

SALL MHN. (1995). Cell death by apoptosis following anticancer
drug treatment in vitro. Int. J. Oncol., 6, 585-593.

ISHIDA R, MIKI T, NARITA T, YUI R, SATO M, UTSUMI KR,

TANABE K AND ANDOH T. (1991). Inhibition of intracellular
topoisomerase II by antitumor bis(2,6-dioxopiperazine) deriva-
tives: mode of cell growth inhibition distinct from that of
cleavable complex-forming type inhibitors. Cancer Res., 51,
4909-4916.

KAUFMANN SH. (1989). Induction of endonucleolytic DNA

cleavage in human acute myelogenous leukaemia cells by
etoposide, camptothecin, and other cytotoxic anticancer drugs:
a cautionary note. Cancer Res., 49, 5870- 5878.

LI CJ, AVERBOUKH L AND PARDEE AB. (1993). ,B-Lapachone, a

novel DNA topoisomerase I inhibitor with a mode of action
different from camptothecin. J. Biol. Chem., 268, 22463-22468.

MARKOVITS J, LARSEN AK, SEGAL-BENDIRDJIAN E, FOSSE P,

SAUCIER J.-M, GAZIT A, LEVITZKI A, UMEZAWA K AND
JACQUEMIN-SABLON. (1994). Inhibition of DNA topoisome-
rase I and II and induction of apoptosis by erbstatin and
tyrphostin derivatives. Biochem. Pharmacol., 48, 549 - 560.

MEIKLE I, CUMMINGS J, MACPHERSON JS, HADFIELD JA AND

SMYTH JF. (1995a). Biochemistry of topoisomerase I and II
inhibition by anthracenyl - amino acid conjugates. Biochem.
Pharmacol., 49, 1747- 1757.

MEIKLE I, CUMMINGS J, MACPHERSON JS, RITCHIE AA AND

SMYTH JF. (1995b). In vitro and in vivo activity of anthracenyl-
amino acid conjugates against human lung, colon and ovarian
cancer. (manuscript submitted for publication).

NELSON EM, TEWEY KM AND LIU LF. (1984). Mechanism of

antitumor drug action: poisoning of mammalian DNA topoi-
somerase II on DNA by 4'-(9-acridinylamino)methanesulfon-m-
anisidide. Proc. Natl Acad. Sci. USA, 81, 1361- 1365.

OBERHAMMER F, FRITSCH G, SCHMIED M, PAVELKA M, PRINTZ

D, PURCHIO T, LASSMANN H AND SCHULTE-HERMANN R.
(1993a). Condensation of the chromatin at the membrane of an
apoptotic nucleus is not associated with activation of an
endonuclease. J. Cell Sci., 104, 317- 326.

OBERHAMMER F, WILSON JW, DIVE C, MORRIS ID, HICKMAN JA,

WAKELING AE, WALKER PR AND SIKORSKA M. (1993b).
Apoptotic death in epithelial cells: cleavage of DNA to 300 and/
or 50 kb fragments prior to or in the absence of internucleosomal
fragmentation. EMBO J., 12, 3679-3684.

ONISHI Y, AZUMA Y, SATO Y, MIZUNO Y, TADAKUMA T AND

KIZAKI H. (1993). Topoisomerase inhibitors induce apoptosis in
thymocytes. Biochim. Biophys. Acta, 1175, 147- 154.

ONISHI Y, AZUMA Y AND KIZAKI H. (1994). Bis(2,6-dioxopiper-

azine) derivatives, topoisomerase II inhibitors which do not form
a DNA cleavable complex, induce thymocyte apoptosis. Biochem.
Mol. Biol. Intl., 32, 115 - 122.

PERMANA PA, SNAPKA RM, SHEN LL, CHU DTW, CLEMENT JJ

AND PLATTNER JJ. (1994). Quinobenoxazines: a class of novel
antitumor quinolones and potent mammalian DNA topoisome-
rase II catalytic inhibitors. Biochemistry, 33, 11333- 11339.

ROY C, BROWN DL, LITTLE JE, VALENTINE BK, WALKER PR,

SIKORSKA M, LEBLANC J AND CHALY N. (1992). The
topoisomerase II inhibitor teniposide (VM-26) induces apoptosis
in unstimulated mature murine lymphocytes. Exp. Cell Res., 200,
4 16-424.

SCHNEIDER E, HSIANG Y.-H AND LIU LF. ( 1990). DNA

topoisomerase as anticancer drug targets. Adv. Pharmacol., 21,
149- 183.

References

ARENDS MJ, MORRIS RG AND WYLLIE AH. (1990). Apoptosis. The

role of the endonuclease. Am. J. Pathol., 136, 593 -608.

BERTRAND R, KERRIGAN D, SARANG M AND POMMIER Y. (1991).

Cell death induced by topoisomerase inhibitors. Role of calcium
in mammalian cells. Biochem. Pharmacol., 42, 77 - 85.

BICKNELL GR, SNOWDEN RT AND COHEN GM. (1994). Formation

of high molecular mass DNA fragments is a marker of apoptosis
in the human leukaemic cell line, U937. J. Cell Sci., 107, 2483-
2489.

CHEN AY AND LIU LF. (1994). DNA topoisomerase: essential

enzymes and lethal targets. Annu. Rev. Pharmacol. Toxicol., 34,
191 -218.

COHEN GM, SUN XM, SNOWDEN RT, DINSDALE D AND

SKILLETER DN. (1992). Key morphological features of apoptosis
may occur in the absence of internucleosomal DNA fragmenta-
tion. Biochem. J., 286, 331-334.

COHEN GM, SUN XM, FEARNHEAD H, MACFARLANE M, BROWN

DG, SNOWDEN RT AND DINSDALE D. (1994). Formation of large
molecular weight fragments of DNA is a key committed step of
apoptosis in thymocytes. J. Immunol., 153, 507-516.

COHEN JJ AND DUKE RC. (1984). Glucocorticoid activation of a

calcium-dependent endonuclease in thymocyte nuclei leads to cell
death. J. Immunol., 132, 38-42.

CORBETT AH AND OSHEROFF N. (1993). When good enzymes go

bad: conversion of topoisomerase II to a cellular toxin by
antineoplastic drugs. Chem. Res. Toxicol., 6, 585- 597.

CUMMINGS J AND SMYTH JF. (1993). DNA topoisomerase I and II

as targets for rational design of new anticancer drugs. Ann.
Oncol., 4, 533-543.

CUMMINGS J, MACPHERSON JS, HICKSON ID, DAVIES SL,

MINCHER DJ AND SMYTH JF. (1994). Drug development of
anthracenyl -amino acid/peptide topoisomerase I and II inhibi-
tors which circumvent drug resistance. Ann. Oncol., 5, (suppl. 5),
186.

CUMMINGS J, MACPHERSON JS, MEIKLE I AND SMYTH JF. (1995a).

Development of anthracenyl- amino acid/peptide topoisomerase
I and II inhibitors which circumvent drug resistance. Biochem.
Pharmacol., (in press).

CUMMINGS J, SUMMER AT, SLAVOTINEK A, MEIKLE I, MAC-

PHERSON JS AND SMYTH JF. (1995b). Cytogenetic evaluation of
the mechanism of cell death induced by the novel anthracenyl-
amino acid topoisomerase II catalytic inhibitor NU/ICRF 500.
Mutat. Res., 344, 55-62.

DEL BINO G AND DARZYNKIEWICZ Z. (1991). Camptothecin,

teniposide, or 4'-(9-acridinylamino)-3-methanesulfon-m-anisi-
dide, but not mitoxantrone or doxorubicin, induces degradation
of nuclear DNA in the S phase of HL60 cells. Cancer Res., 51,
1165-1169.

EARNSHAW WC. (1995). Nuclear changes in apoptosis. Curr. Opin.

Cell Biol., 7, 337-343.

FILLIPSKI J, LEBLANC J, YOUDALE T, SIKORSKA M AND WALKER

PR. (1990). Periodicity of DNA folding in higher order chromatin
structures. EMBO J., 9, 1319- 1327.

GULLIYA KS, FRANCK B, SCHNEIDER U, SHARMA R, ARNOLD L

AND MATTHEWS JL. (1994). Topoisomerase II-dependent novel
antitumour compounds merocil and merodantoin induce apop-
tosis in Daudi cells. Anticancer Drugs, 5, 557- 566.

GEWIRTZ DA. (1991). Does bulk damage to DNA explain the

cytotoxic effects of topoisomerase inhibitors? Biochem. Pharma-
col., 42, 2253-2258.

HICKMAN JA. (1992). Apoptosis induced by anticancer drugs.

Cancer Metastasis Rev., 11, 121 - 139.

HSIANG Y.-H, HERTZBERG R, HECHT S AND LIU LF. (1985).

Camptothecin induces protein-linked DNA breaks via mamma-
lian DNA topoisomerase I. J. Biol. Chem., 260, 14873- 14878.

HUSCHTSCHA LI, BARTIER WA, MALMSTROM A AND TATTER-

SALL MHN. (1995). Cell death by apoptosis following anticancer
drug treatment in vitro. Int. J. Oncol., 6, 585-593.

ISHIDA R, MIKI T, NARITA T, YUI R, SATO M, UTSUMI KR,

TANABE K AND ANDOH T. (1991). Inhibition of intracellular
topoisomerase II by antitumor bis(2,6-dioxopiperazine) deriva-
tives: mode of cell growth inhibition distinct from that of
cleavable complex-forming type inhibitors. Cancer Res., 51,
4909-4916.

KAUFMANN SH. (1989). Induction of endonucleolytic DNA

cleavage in human acute myelogenous leukaemia cells by
etoposide, camptothecin, and other cytotoxic anticancer drugs:
a cautionary note. Cancer Res., 49, 5870- 5878.

LI CJ, AVERBOUKH L AND PARDEE AB. (1993). ,B-Lapachone, a

novel DNA topoisomerase I inhibitor with a mode of action
different from camptothecin. J. Biol. Chem., 268, 22463-22468.

MARKOVITS J, LARSEN AK, SEGAL-BENDIRDJIAN E, FOSSE P,

SAUCIER J.-M, GAZIT A, LEVITZKI A, UMEZAWA K AND
JACQUEMIN-SABLON. (1994). Inhibition of DNA topoisome-
rase I and II and induction of apoptosis by erbstatin and
tyrphostin derivatives. Biochem. Pharmacol., 48, 549 - 560.

MEIKLE I, CUMMINGS J, MACPHERSON JS, HADFIELD JA AND

SMYTH JF. (1995a). Biochemistry of topoisomerase I and II
inhibition by anthracenyl - amino acid conjugates. Biochem.
Pharmacol., 49, 1747- 1757.

MEIKLE I, CUMMINGS J, MACPHERSON JS, RITCHIE AA AND

SMYTH JF. (1995b). In vitro and in vivo activity of anthracenyl-
amino acid conjugates against human lung, colon and ovarian
cancer. (manuscript submitted for publication).

NELSON EM, TEWEY KM AND LIU LF. (1984). Mechanism of

antitumor drug action: poisoning of mammalian DNA topoi-
somerase II on DNA by 4'-(9-acridinylamino)methanesulfon-m-
anisidide. Proc. Natl Acad. Sci. USA, 81, 1361- 1365.

OBERHAMMER F, FRITSCH G, SCHMIED M, PAVELKA M, PRINTZ

D, PURCHIO T, LASSMANN H AND SCHULTE-HERMANN R.
(1993a). Condensation of the chromatin at the membrane of an
apoptotic nucleus is not associated with activation of an
endonuclease. J. Cell Sci., 104, 317- 326.

OBERHAMMER F, WILSON JW, DIVE C, MORRIS ID, HICKMAN JA,

WAKELING AE, WALKER PR AND SIKORSKA M. (1993b).
Apoptotic death in epithelial cells: cleavage of DNA to 300 and/
or 50 kb fragments prior to or in the absence of internucleosomal
fragmentation. EMBO J., 12, 3679-3684.

ONISHI Y, AZUMA Y, SATO Y, MIZUNO Y, TADAKUMA T AND

KIZAKI H. (1993). Topoisomerase inhibitors induce apoptosis in
thymocytes. Biochim. Biophys. Acta, 1175, 147- 154.

ONISHI Y, AZUMA Y AND KIZAKI H. (1994). Bis(2,6-dioxopiper-

azine) derivatives, topoisomerase II inhibitors which do not form
a DNA cleavable complex, induce thymocyte apoptosis. Biochem.
Mol. Biol. Intl., 32, 115 - 122.

PERMANA PA, SNAPKA RM, SHEN LL, CHU DTW, CLEMENT JJ

AND PLATTNER JJ. (1994). Quinobenoxazines: a class of novel
antitumor quinolones and potent mammalian DNA topoisome-
rase II catalytic inhibitors. Biochemistry, 33, 11333- 11339.

ROY C, BROWN DL, LITTLE JE, VALENTINE BK, WALKER PR,

SIKORSKA M, LEBLANC J AND CHALY N. (1992). The
topoisomerase II inhibitor teniposide (VM-26) induces apoptosis
in unstimulated mature murine lymphocytes. Exp. Cell Res., 200,
416-424.

SCHNEIDER E, HSIANG Y.-H AND LIU LF. (1990). DNA

topoisomerase as anticancer drug targets. Adv. Pharmacol., 21,
149-183.

Induction of apoptosis by NU/ICRF 505
I Meikle et al

SOLARY E, BERTRAND R, KOHN KW AND POMMIER Y. (1993).

Differential induction of apoptosis in undifferentiated and
differentiated HL60 cells by DNA topoisomerase I and II
inhibitors. Blood, 81, 1359- 1368.

SUN XM, SNOWDEN RT, SKILLETER DN, DINSDALE D, ORMEROD

MG AND COHEN GM. (1994). Changes in nuclear chromatin
precede internucleosomal DNA cleavage in the induction of
apoptosis by etoposide. Biochem. Pharmacol., 47, 187- 195.

WALKER PR, SMITH C, YOUDALE T, LEBLANC J, WHITFIELD JF

AND SIKORSKA M. (1991). Topoisomerase TI-reactive chemother-
apeutic drugs induce apoptosis in thymocytes. Cancer Res., 51,
1078- 1085.

WANG JC. (1985). DNA topoisomerases. Annu. Rev. Biochem., 54,

665 - 697.

WYLLIE AH. (1980). Glucocorticoid induced thymocyte apoptosis is

associated with endogenous endonuclease activation. Nature, 284,
555 - 556.

WYLLIE AH, MORRIS RG, SMITH AL AND DUNLOP D. (1984).

Chromatin cleavage in apoptosis: association with condensed
chromatin morphology and dependence on macromolecular
synthesis. J. Pathol., 142, 67 - 77.

ZWELLING LA. (1989). Topoisomerase II as a target of anti-

leukaemic drugs: a review of controversial areas. Hum. Pathol.,
3, 101-112.

				


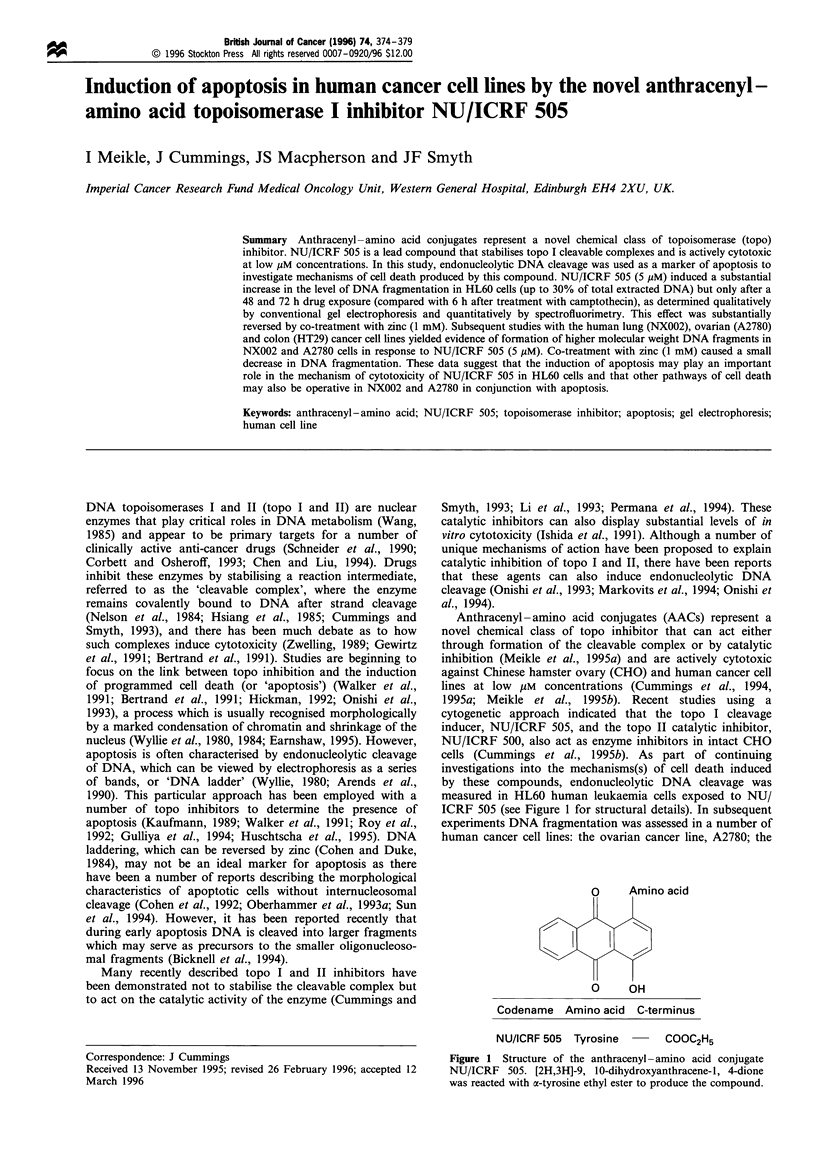

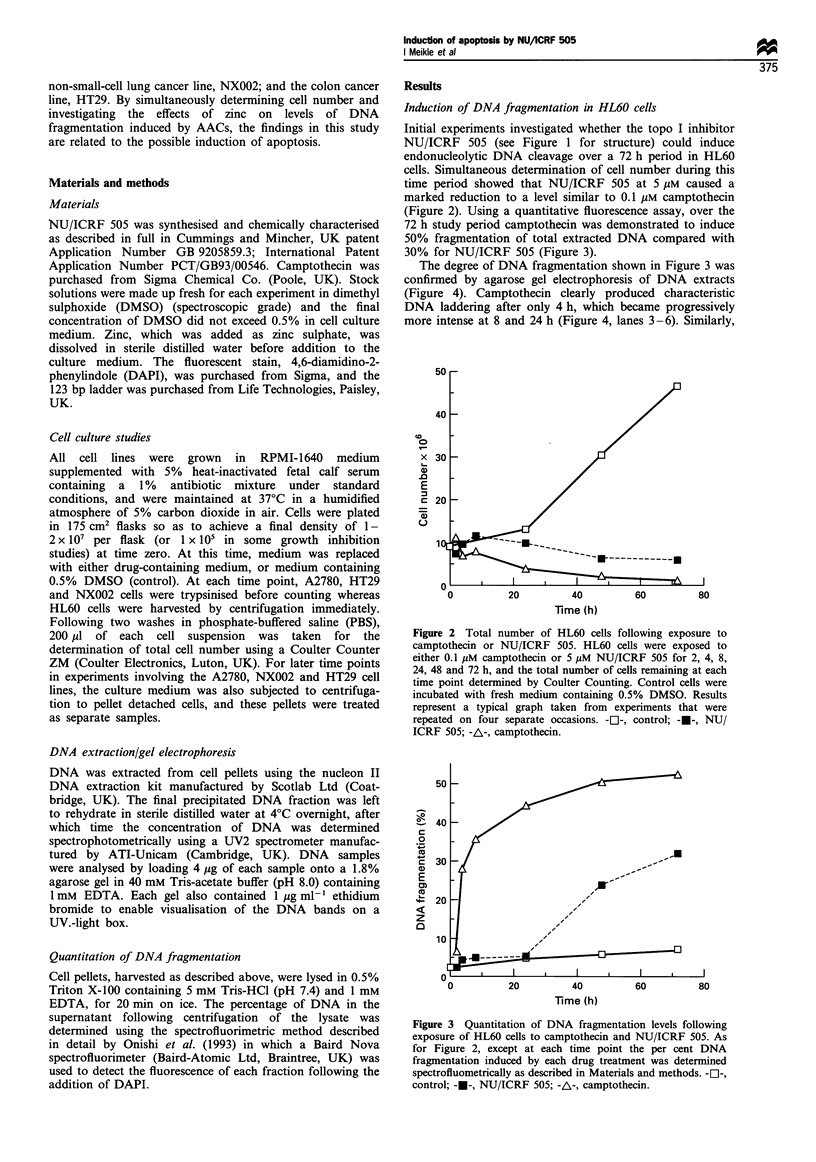

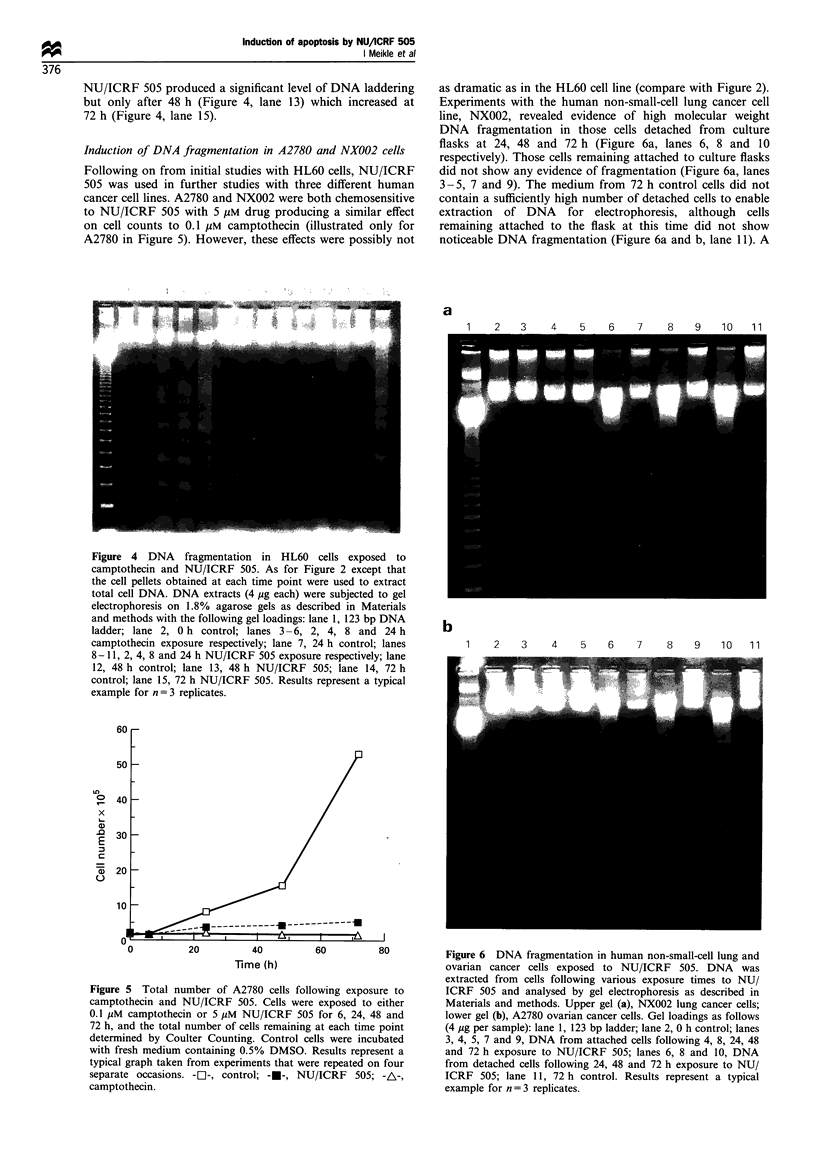

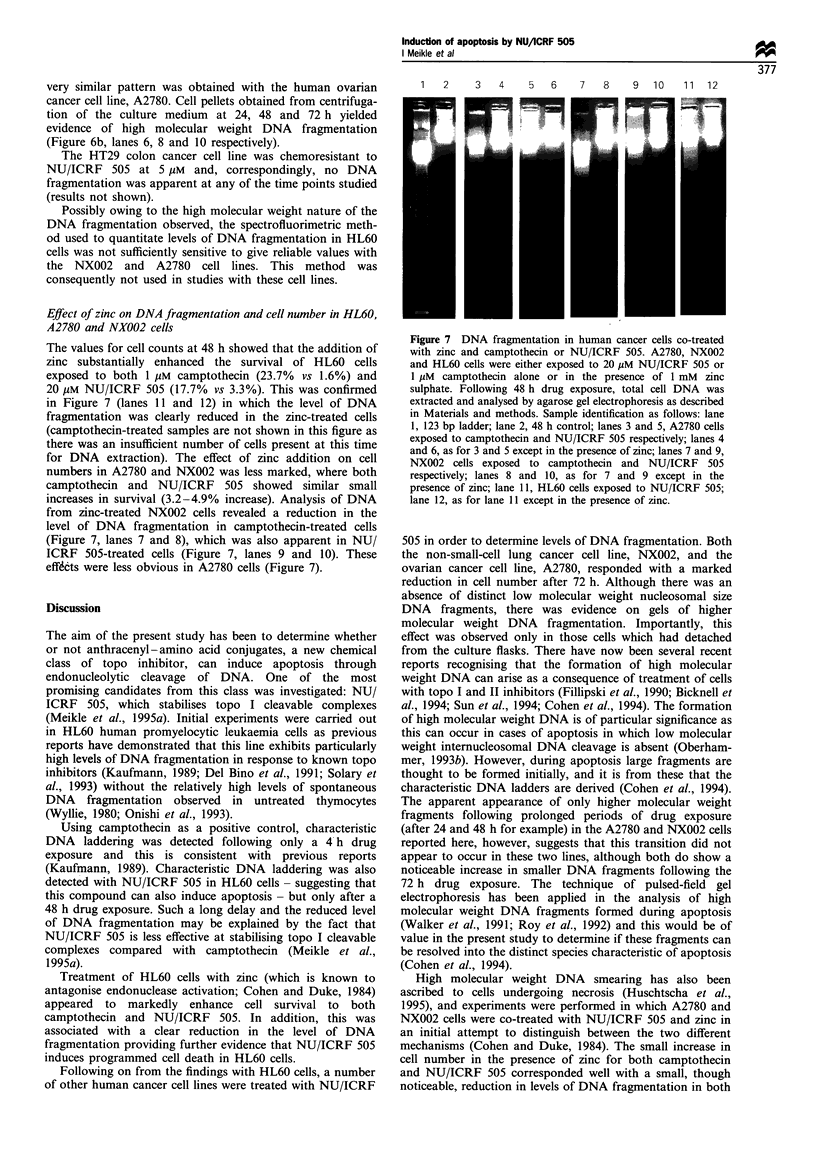

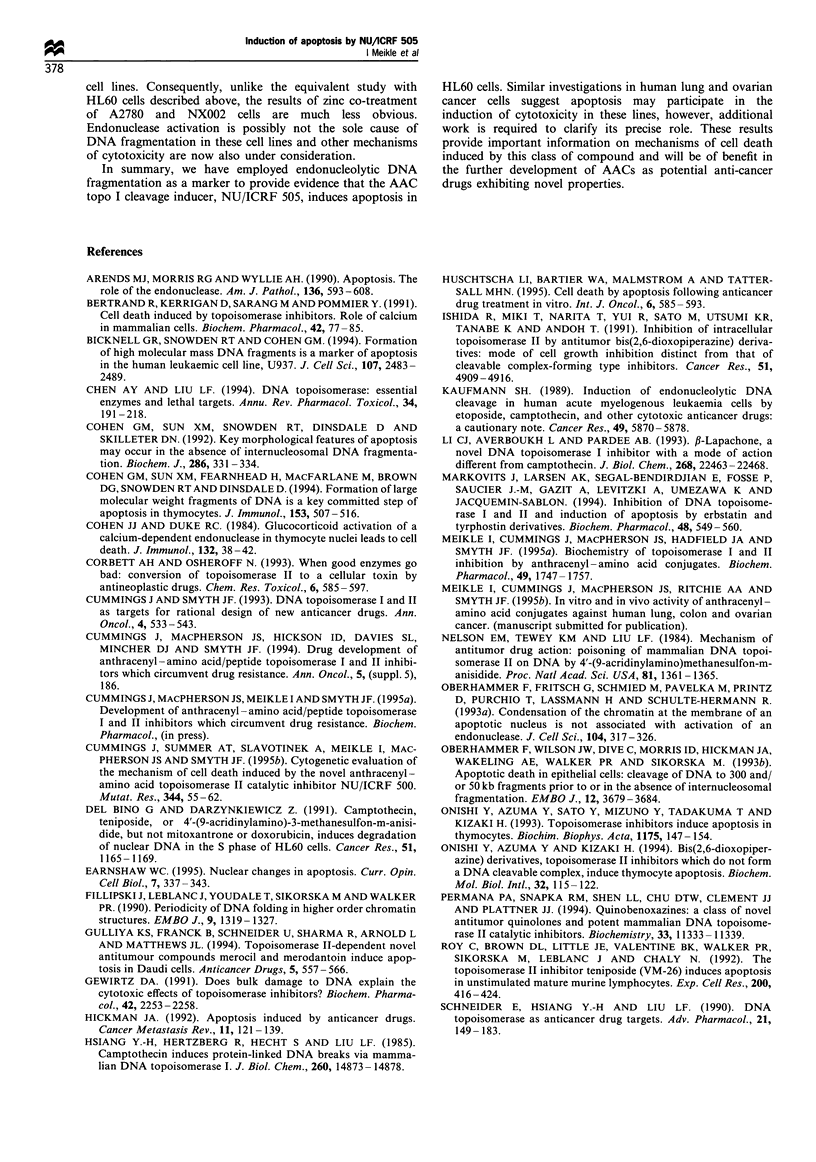

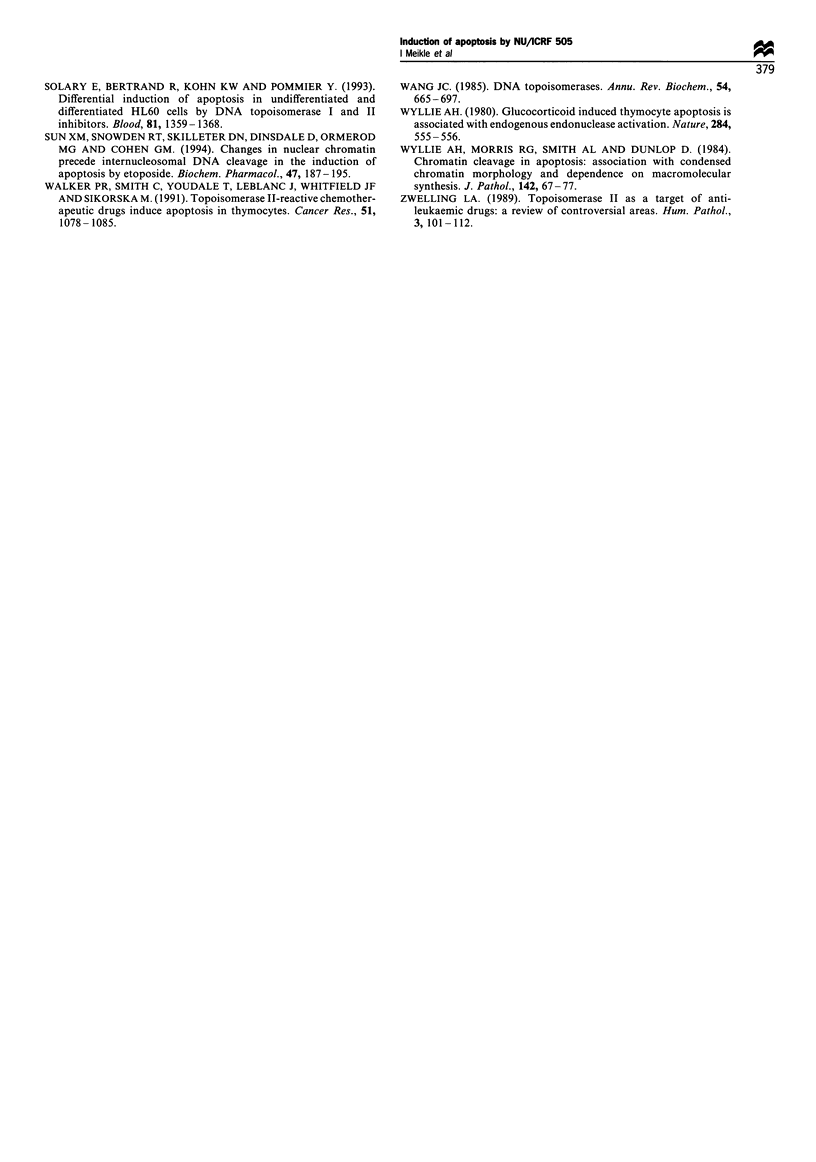

